# Usability Evaluation of an Electrically Powered Orthopedic Exerciser: Focus Group Interview and Satisfaction Survey Study

**DOI:** 10.2196/60607

**Published:** 2025-05-30

**Authors:** Seojin Hong, Hyun Choi, Hyosun Kweon

**Affiliations:** 1 Department of Clinical Rehabilitation Research, Rehabilitation Research Institute, Korea National Rehabilitation Center, 58 Samgaksan-ro, Gangbuk-gu, Seoul, 01022, Republic of Korea, 82 02-901-1966; 2 Department of Healthcare and Public Health, Rehabilitation Research Institute, Korea National Rehabilitation Center, Seoul, Republic of Korea

**Keywords:** usability evaluation, usability, electrically powered orthopedic exerciser, focus group interview, satisfaction survey

## Abstract

**Background:**

Musculoskeletal disorders significantly impair physical function and quality of life, necessitating systematic rehabilitation. Electrically powered orthopedic exercisers, such as continuous passive motion devices, are widely used to enhance joint mobility and muscle recovery. However, existing devices often lack advanced functionalities and user-specific adaptability, limiting their effectiveness. To address these shortcomings, the Rebless Pro was developed as a novel device supporting active and passive exercises with personalized treatment programs.

**Objective:**

This study aimed to conduct a formative usability evaluation of the Rebless Pro prototype using focus group interviews (FGIs) and satisfaction surveys with health care professionals specializing in rehabilitation medicine. The goal was to identify areas for improvement to enhance the safety, usability, and information clarity of the device.

**Methods:**

Usability evaluation was performed at the National Rehabilitation Center with 10 participants (5 physiatrists and 5 physical therapists) who had prior experience using similar devices. FGIs were conducted to collect qualitative insights into user experiences, while satisfaction surveys provided quantitative data on ease of use of the user interface and identifiability and understanding of information. Data collection focused on identifying risk factors and usability challenges.

**Results:**

Three key areas for improvement were identified: (1) product upgrades to ensure patient safety, including adjustments to exercise speed and resistance; (2) hardware and software improvements to improve usability, including adjustments to the location of the emergency button and improvements to the graphical user interface elements; and (3) improvements to the user manual, including detailed contraindications, patient criteria, and clearer operating instructions. Although the mean score of physiatrists (mean 4.463, SD 0.298) was higher than that of physical therapists (mean 4.114, SD 0.829) in terms of the ease of use of the user interface, the difference was not statistically significant (*P*=.69). Similarly, in the category of identifiability and understanding of information, higher scores were again reported by physiatrists (mean score 4.467, SD 0.506) than by physical therapists (mean 3.733, SD 0.894), but this difference was also not statistically significant (*P*=.22).

**Conclusions:**

Usability evaluation provided actionable insights into improving the Rebless Pro’s safety, usability, and information clarity. To further refine the device, iterative usability evaluations involving both health care professionals and patients are recommended. These efforts are expected to contribute to the development of a safe, effective, and user-friendly electrically powered orthopedic exerciser suitable for commercialization.

## Introduction

### Background

Musculoskeletal (MSK) disorders, resulting from diseases or injuries affecting muscles, tendons, ligaments, and bones, are a major cause of reduced physical function and quality of life [1]. Patients with MSK disorders often experience difficulties in performing independent daily activities due to reduced joint mobility, weakened muscle strength, and chronic pain, necessitating systematic and continuous rehabilitation therapy [2]. In clinical rehabilitation, electrically powered orthopedic exercisers play a crucial role in facilitating joint tissue regeneration and muscle strength recovery [3].

Continuous passive motion (CPM) devices are representative examples of exercisers; they are widely used to prevent joint stiffness and promote tissue healing, particularly after orthopedic surgeries [4,5]. However, despite their therapeutic benefits, conventional CPM devices have several limitations that hinder optimal rehabilitation outcomes. First, they typically provide only passive range of motion (ROM) exercises without accommodating more dynamic modes, such as active-assisted or resisted exercises, which are essential for patients requiring progressive muscle strengthening or functional restoration [6,7]. Second, these devices lack user-specific customization features. Most CPM units operate on fixed protocols that do not account for individual differences in pain tolerance, ROM progression, or rehabilitation goals [8,9]. As a result, patients may experience discomfort or disengagement due to inappropriate exercise intensity or duration. Third, many CPM devices are ergonomically unsuitable for certain populations, such as individuals with obesity, severe joint deformities, or an inability to maintain a supine position. Since most CPM devices are designed for supine use, they may also limit usability and patient compliance in nonhospital settings [10,11].

To address these issues, alternative models, such as sitting-type CPM devices, have been developed. These devices allow patients to perform rehabilitation exercises in a seated position, which can enhance comfort, accessibility, and safety. Some studies have reported that sitting-type CPM devices offer comparable efficacy to conventional models while improving patient satisfaction [9]. Nevertheless, these alternatives are still relatively underused in clinical practice, partly due to limited availability and familiarity among clinicians.

In response to the need for more adaptive and patient-centered solutions, the Rebless Pro was developed as a next-generation orthopedic exerciser supporting both passive and active-assisted exercises. The device was designed to allow personalized treatment protocols and improve usability in various clinical settings.

### Electrically Powered Orthopedic Exerciser

**Figure 1.**
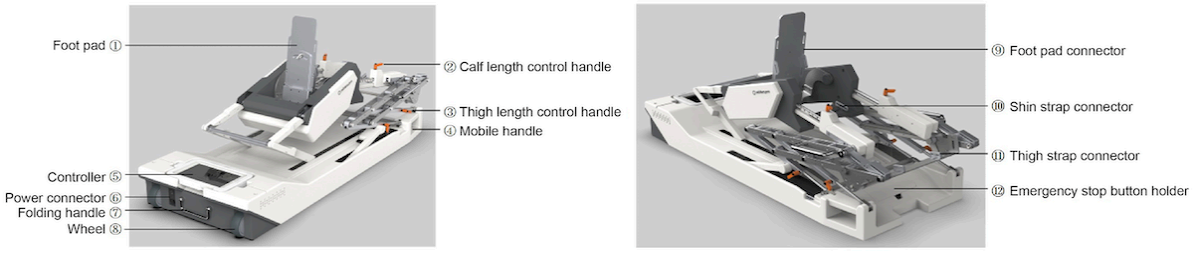
Electrically powered orthopedic exerciser.

The Rebless Pro (H Robotics Inc) is an electrically powered orthopedic exerciser developed to support full ROM exercises and facilitate active and passive rehabilitation of the knee and ankle in individuals with neurological or MSK disorders (Figure 1). During the initial design phase, user requirements were collected from clinicians to inform the development of device functionalities and interface elements. Currently in its prototype stage, the Rebless Pro comprises a main unit and a tablet-based controller operated via the Rebless Pro app.

The app features an intuitive graphical user interface (UI) and is structured around five core functional modules: (1) passive ROM, which enables therapist-initiated joint mobilization; (2) active-assisted ROM, providing adjustable support for patient-initiated movement; (3) evaluation ROM measurements, allowing objective tracking of joint range; (4) resisted ROM, designed to improve muscular strength and motor control through applied resistance; and (5) interactive custom setup, which enables therapists to personalize treatment protocols according to patient-specific needs.

The device operation workflow begins with patient registration and profile setup. Therapists select exercise modes and set individualized parameters—such as ROM extent, resistance, and assistance levels—based on therapeutic goals. During sessions, the app provides real-time feedback on joint movement, resistance, and exercise performance. Upon completion, session data are automatically stored, supporting longitudinal progress monitoring and outcome evaluation.

The Rebless Pro is intended for use by physical therapists and physiatrists, with the primary patient population being individuals with neurological or MSK impairments, including lower-extremity muscle paralysis or joint contractures, as well as people undergoing postsurgical rehabilitation. These users often require guided motor recovery and muscle strengthening to improve functional outcomes.

### Usability Evaluation

Usability issues in medical devices can directly affect patient safety and treatment outcomes, often arising from deficiencies in UI design [12]. To mitigate such risks, the International Electrotechnical Commission (IEC) introduced the IEC 62366 standard, which emphasizes the integration of usability engineering throughout the device development process, distinguishing between formative and summative evaluations [13-15]. Formative evaluations, conducted during the early stages of design, aim to identify UI-related strengths, weaknesses, and potential use errors. These evaluations are iterative and typically involve direct input from intended users to guide design refinement [16], such as from focus group interviews (FGIs), which are widely used during the formative phase to collect user feedback on prototype usability [17,18].

This study conducted a formative usability evaluation of the Rebless Pro, which is currently in its implementation phase. Using FGIs and user satisfaction surveys with rehabilitation medicine professionals—the device’s intended users—this study aimed to identify areas for improvement to enhance the safety and convenience of the device, in accordance with IEC 62366.

## Methods

In this study, FGIs and satisfaction surveys were conducted to collect quantitative and qualitative data on an electrically powered orthopedic exerciser.

### Ethical Considerations

This study was approved by the institutional review board of the National Rehabilitation Center (NRC; NRC-2023-03-020). All participants provided voluntary consent to participate after receiving a comprehensive explanation of the study objectives and methods. No compensation was provided for participating in the study. For the protection of personal information and the confidentiality of the participants, all data were anonymized.

### Participants

The evaluation included 10 NRC-affiliated individuals, including 5 physiatrists and 5 physical therapists, who were recruited through peer referral and a bulletin board announcement at the NRC Usability Testbed.

The inclusion criteria were being a licensed physical medicine and rehabilitation or physical therapist with experience usingelectrically powered orthopedic exercisers and sufficient proficiency in Korean and English to comprehend software terminology. Individuals lacking experience in electrically powered orthopedic exercisers were excluded from the study. The participants provided voluntary consent after receiving a comprehensive explanation of the study objectives, methods, and procedures. All participants had at least 1 year of clinical experience using electrically powered orthopedic exercisers, such as CPM devices. The full formative assessment process, including the FGIs, was conducted separately for the physiatrist group and the physical therapist group.

### Usability Evaluation Procedure

A usability evaluation was conducted at the NRC Usability Testbed. The evaluation was carried out in 2 separate sessions, one each for the physiatrist group and the physical therapist group, with each session lasting 90 minutes. The evaluator provided an introduction to the assessment to familiarize participants who may have been unfamiliar with usability evaluations, explaining the objectives, methods, and procedures of the product evaluation. Following this, participants were required to confirm their understanding of the evaluation details, after which informed consent was obtained for voluntary participation as well as for video and audio recordings. The FGIs commenced with questions regarding the general characteristics of the participants. Subsequently, the evaluator confirmed that the participants had reviewed the relevant information on the Rebless Pro and inquired about their prior experiences with similar devices, including the specific names of those devices. A demonstration of the use scenarios (Multimedia Appendix 1) was then conducted in conjunction with the FGIs using the Rebless Pro. Upon completion of the interviews, a satisfaction survey was administered to gather participants’ feedback.

**Figure 2.**
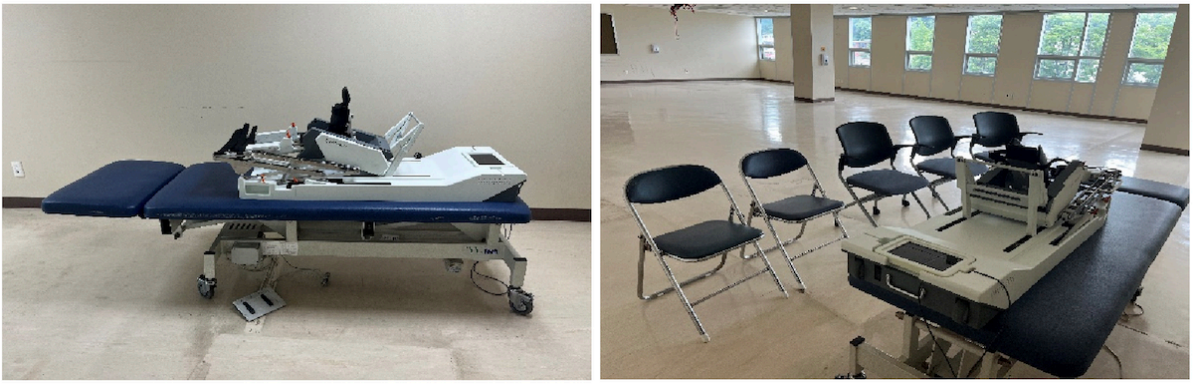
Evaluation environment.

**Figure 3.**
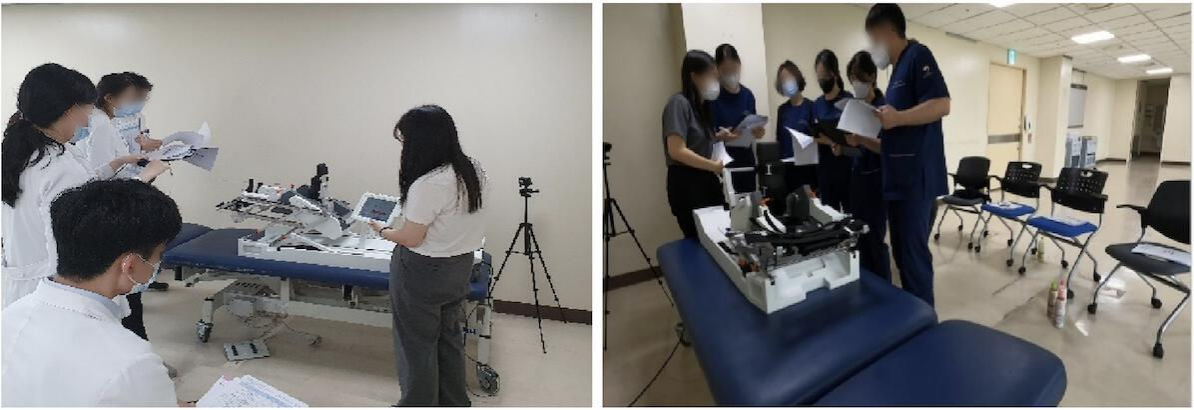
Scenes of the evaluation.

The usability evaluations were conducted in environments that closely resembled real-world use scenarios; such environments have been reported to accurately capture user experience and satisfaction, thereby playing a pivotal role in enhancing the commercial viability of medical devices [19]. In alignment with past findings, this study implemented formative evaluations within a simulated environment designed to replicate actual use conditions. The usability evaluations included a simulated physical therapy environment in which the Rebless Pro was used; the device was set up on a Bobath table (Figure 2). The luminous intensity, temperature, and relative humidity of the evaluation sites were measured before usability evaluation. The luminous intensity was 550 (SD 100) lux, temperature was 24ºC (SD 2ºC), relative humidity was 50% (SD 10%), and ambient noise was 50 (SD 5) dBA. Scenes of the evaluation are shown in Figure 3.

### FGI Procedure

FGIs were used as a qualitative data collection method to evaluate the usability of the Rebless Pro prototype. Each FGI session was conducted once per group, with 1 moderator, 1 observer for recording, and 5 participants present in the evaluation space. The moderator posed identical questions to each participant sequentially, ensuring that all participants—from the first to the fifth—had an opportunity to respond. The FGI questionnaire was structured following the guidelines of Krueger and Casey [17], incorporating introductory, transitional, key, and end questions (Multimedia Appendix 2). To gather feedback on the Rebless Pro, product demonstrations were conducted according to predefined use scenarios. Scenarios for 8 tasks were designed based on the user manual. Participants observed the device during these demonstrations and provided insights into predictable or identified risk factors and areas for UI improvement. The core questions focused on collecting participants’ opinions regarding the UI of the Rebless Pro after observing its functionality and use. Additionally, participants offered suggestions for mitigating risks and improving usability based on their observations during the product demonstrations. Content analysis was conducted on the data obtained from the FGIs [20,21]. Microsoft Excel was used throughout the processes of data transcription, coding, and thematic derivation.

### Satisfaction Surveys

After the FGIs, satisfaction surveys for the Rebless Pro were conducted to collect quantitative data. The satisfaction survey consisted of 51 items on the ease of use of the UI (Cronbach α=0.987) and 3 items on identifiability and understanding of information (Cronbach α=0.617). The survey items were rated on a 5-point Likert scale ranging from 1 (very difficult) to 5 (very easy). The difference in satisfaction between the physiatrist group and the physical therapist group was analyzed using the Mann-Whitney *U* test in SPSS (version 25; IBM Corp). The satisfaction survey results are presented as means and SDs for each item by group (MultimediaAppendices 3  4).

## Results

### Participant Characteristics

In the physiatrist group, clinical experience ranged from 1 year and 3 months to 2 years and 3 months. Experience in using similar medical devices ranged from 3 months to more than 1 year. In the physical therapist group, clinical experience ranged from 7 to 28 years, and experience using similar medical devices was more than 1 year for all members (Table 1). All members of both groups had experience using electrically powered orthopedic exercisers, such as MOTOmed (RECK-Technik GmbH) and other CPM devices, but all were using the Rebless Pro for the first time.

**Table 1.** General participant characteristics.

Number	Sex	Age (years)	Experience	Occupation	Experience using similar medical devices	Similar medical device
1	Female	29	2 yr 3 mo	Physiatrist	1 yr, >once a day	MOTOmed
2	Female	27	2 yr 3 mo	Physiatrist	1 mo, once a day	CPM^a^ device
3	Male	26	2 yr 3 mo	Physiatrist	6 mo, once a day	MOTOmed
4	Female	28	1 yr 3 mo	Physiatrist	4 mo, once a day	MOTOmed
5	Male	25	1 yr 3 mo	Physiatrist	3 mo, twice a day	MOTOmed
6	Female	51	28 yr 10 mo	Physical therapist	1 yr, 3 times a day	MOTOmed
7	Male	40	14 yr	Physical therapist	1 yr, once a day	MOTOmed
8	Female	36	9 yr 10 mo	Physical therapist	1 yr, once a day	MOTOmed
9	Female	32	9 yr 6 mo	Physical therapist	1 yr, once a day	MOTOmed
10	Female	29	7 yr 3 mo	Physical therapist	1 yr, 3 times a day	MOTOmed, CPM device

a CPM: continuous passive motion.

### FGI Results

The FGIs revealed 3 key factors and 7 subfactors representing possible improvements to the Rebless Pro. The three key factors were as follows: (1) product upgrades to ensure safety, (2) hardware and software improvements for convenience of use, and (3) improvement of the manual for better identifiability and understanding of the information (Table 2).

**Table 2.** Outcome of content analysis.

Factor	Suggested solution
1. Product upgrades to ensure safety	Improve the exterior Fasten the device to the table Adjust the motor for exercise speed and resistance
2. Improvements for convenience of use	Improve the hardware user interface Improve the software user interface
3. Improvement to the manual for better identifiability and understanding of the information	Input details about the intended patient group and medical indications Input details about device use and operation

### Product Upgrades to Ensure Safety

The participants prioritized patient safety and suggested the following potential risks of and improvements to the Rebless Pro:

The device should be fastened to the table, but force applied by the therapist should allow it to slide or move.

The exterior requires improvement as the device has a complex structure and accidents may be caused through items getting caught on its exterior.

The device should be secured using an anti-slip pad.

Patients may complain of pain because the speed of the basic exercise may be too fast. The initial position angle should be adjusted while the device is being fitted.

Movement speed needs to be more finely adjusted. The operating exercise speed should be shown on the controller display.

The basic resistance intensity of the device may be too high for the patient to counteract.

There is a high likelihood of errors occurring during ROM measurement, and measurement range errors may occur.

### Hardware and Software Improvements for Convenience of Use

For user convenience and ease of use, the following hardware and software improvements to the Rebless Pro were suggested:

The power (on, off) button should be repositioned.

The emergency stop button should be repositioned, and separate buttons should be provided for the patient and therapist.

The material of the fastening strap where the device is secured to the patient should be changed.

Noise from the motor may interfere with the treatment of other patients. The motor noise should be adjusted.

As this device is provided to the patient by medical staff, the wording of the instructions should be improved to make it easier for patients to understand (instead of using medical terminology).

The information about intensity and stiffness sensitivity is provided only in the manual. It is cumbersome to have to refer to the manual while using the device.

A pop-up window with a summary of each function should be added to the controller display.

Control icons, such as up and down arrows, should be presented more clearly on the display.

### Improvement of the Manual for Better Identifiability and Understanding of the Information

Participants suggested the following improvements to the Rebless Pro manual to allow better identifiability and understanding of information by users:

Detailed information about the contraindications for intended patient groups, stages of life and age groups, specific criteria for applicable patients, and the patient’s position when fitting the device should be provided.

With respect to use of the device, calf/thigh measurement criteria, exercise intensity criteria based on Manual Muscle Test or Modified Ashworth Scale standards, and clear exercise speed criteria should be provided.

### Satisfaction Survey Results

To identify the difference in satisfaction between physiatrists and physical therapists, a Mann-Whitney *U* test was conducted. When comparing the overall ease of use of the UI, the satisfaction of the physiatrists (mean score 4.463, SD 0.298) was higher than that of the physical therapists (mean score 4.114, SD 0.829), but this was not a statistically significant difference (*U*=10.000; *Z*=−0.522; *P*=.69). In addition, when comparing the overall information identifiability, the satisfaction of the physiatrists (mean score 4.467, SD 0.506) was higher than that of the physical therapists (mean score 3.733, SD 0.894), but this was not a statistically significant difference (*U=*6.000; *Z*=−1.375; *P*=.22).

## Discussion

### Principal Results

A formative evaluation is a usability evaluation performed to improve the design of a medical device under development. To gain qualitative and quantitative insights into the course of the development of the Rebless Pro, an electrically powered orthopedic exerciser, we conducted a formative evaluation. This was achieved using FGIs and satisfaction surveys with rehabilitation medicine professionals who had experience using other electrically powered orthopedic exercisers.

FGIs enable individuals to freely express their own experiences and engage in discussions using not only their own knowledge but that of others, thereby providing diverse perspectives and detailed information on a specific topic [17]. Moreover, FGIs are used to identify and analyze expert responses as experts share information and experience in using a particular product or service. Therefore, FGI findings may be used to develop or improve products and services [22].

Motorized medical devices can cause unintended movements by the patient or user, potentially leading to discomfort or injury [23]. Because the operator of the Rebless Pro is a medical professional and the user is a patient, the participants in this study prioritized patient safety and presented opinions on improvement of the exterior structure of the device and upgrades to the device fastening method, exercise speed, and resistance strength. These points suggest that patient and operator safety is crucial when designing a rehabilitation device and that the product should be upgraded to ensure safety.

A previous study concluded that devices using robotic technology should be equipped with at least one emergency stop button that is easily accessible to the user [24], and another study reported that an emergency stop system should be installed to prioritize user and patient safety in the event of device malfunction or user error [12]. On the basis of their own experience using similar medical devices and the product demonstration, the participants in our study indicated the need for the emergency button to be redesigned and repositioned so that it would be more visible and easily accessible to medical staff or patients in the event of an emergency. Consequently, we determined that the emergency stop system of a medical device has a direct impact on the ability to prevent device malfunction and that it plays an important role in ensuring device safety and efficacy.

The Rebless Pro is a novel device currently under research, meaning that all users, including medical staff and patients, can be considered novices in its operation. As the device is primarily operated by medical staff but worn by patients, it is crucial that the UI design and user manual prioritize intuitiveness and accessibility. This ensures that both medical staff and patients can easily learn to operate the device. Consistent with previous findings, simple terms should be used in the manual to facilitate quick and accurate operation, allowing users to interact with the device intuitively and consistently [25].

From an ergonomic perspective, the medical device design should consider not only convenience and usability but also the prevention of possible injuries caused by repeated strain and the physical defects of individual people [26]. The key to CPM devices is their ability to deliver the same movement as the actual human body through human lower-extremity ROM and accurate alignment [27]. As in previous studies, marking the joint ROM and axes on the surface of the medical device was suggested in this study as an area of improvement for electrically powered orthopedic exercisers, considering the individual physical factors of each patient.

The user manual of a medical device is important not only for usability but also for identifying and understanding information [28]. The user manual explains device operation, maintenance, and troubleshooting, which can potentially affect patient safety [29]. The participants in this study also requested specific information regarding specific operating standards and clear information about the intended patient groups, including contraindications and applicable age. Based on previous and the present studies, the development of a high-quality user manual is essential for the effective use of the device.

The high average scores (≥4) for ease of use of the UI were likely influenced by the fact that participants assessed the device during a controlled product demonstration rather than real clinical use. The findings also showed that the mean satisfaction for information identifiability and understanding of information were lower in the physical therapist group than in the physiatrist group. In rehabilitation medicine, physiatrists and physical therapists form a collaborative, multidisciplinary team; however, physiatrists are responsible for establishing the overall treatment plan, whereas physical therapists specialize in assessing motor problems and providing rehabilitation training [30-32]. The satisfaction scores may have been lower for physical therapists than for physiatrists because the physical therapists assigned scores for identification and understanding of the Rebless Pro on the basis of their own experience. These findings confirm that when developing a medical device, the intended users should be clearly defined so that they can satisfy the information identifiability and understanding requirements.

Because continuous formative evaluations of improved medical devices are important to ensure their efficacy [33], repeated formative evaluations are required for the commercialization of our high-quality electrically powered orthopedic exerciser. Improving the UI to reduce errors that may cause serious harm is recommended to enhance device efficacy and safety.

### Limitations

Owing to the nature of rehabilitation devices, both medical staff who operate the device and patients who receive therapy are considered users. However, this study focused on evaluating the usability of the Rebless Pro prototype from the perspective of rehabilitation professionals, specifically examining tasks performed by medical staff, such as operating the tablet-based controller and assisting patients in wearing the device. Although we did not include patients as direct participants, we obtained valuable insights from health care professionals specializing in rehabilitation medicine, including perspectives from the patient’s viewpoint during the evaluation process. Also, standardized tools such as the System Usability Scale were not used for quantitative data collection in this study. To ensure further refinement of the device through iterative formative evaluations, future studies should incorporate standardized tools to collect quantitative data.

### Conclusions

If improvements are made to the safety of the electrically powered orthopedic exerciser, the identifiability and comprehensibility of information related to it, and its ease of use based on identified risk factors and areas for UI enhancement, the device can be refined to better meet user needs. This study primarily identified physiatrists and physical therapists as key user groups for evaluating the electrically powered orthopedic exerciser, given their expertise in device operation and patient safety. Future evaluations should further integrate patient feedback to ensure usability and safety from both clinical and end-user perspectives. Iterative formative evaluations involving these specific user groups, alongside the systematic implementation of necessary modifications, will facilitate a summative evaluation of the final product. These efforts are expected to contribute to the commercialization of an electrically powered orthopedic exerciser that prioritizes both safety and usability for its intended users.

## Supplementary material

10.2196/60607 Multimedia Appendix 1 Use scenarios for rehabilitation medical staff.

10.2196/60607 Multimedia Appendix 2 Question guide for focus group interviews.

10.2196/60607 Multimedia Appendix 3 Survey results on ease of use of the user interface.

10.2196/60607 Multimedia Appendix 4 Survey results on identifiability and understanding of information.

## Abbreviations

CPM: continuous passive motion
FGI: focus group interview
IEC: International Electrotechnical Commission
MSK: musculoskeletal
NRC: National Rehabilitation Center
ROM: range of motion
UI: user interface
