# Supplementary material for: Usability Evaluation of an Electrically Powered Orthopedic Exerciser: Focus Group Interview and Satisfaction Survey Study
Source: JMIR Hum Factors. 2025 May 30;12:e60607. doi: 10.2196/60607 (PMC12143852; doi:10.2196/60607)
Supplement: Multimedia Appendix 1 [file humanfactors-v12-e60607-s001.docx]

Appendix 1. Use scenarios for rehabilitation medical staff

| Use scenarios | Task description |
| --- | --- |
| **Task 1. Turn on the power** | |
| Sub-task 1 | Connect the power cable plug to the main unit connector and press the “ON” sign on the power switch to turn on the device |
| Sub-task 2 | Check the controller power and select “Start exercise now” |
| Sub-task 3 | Select the exercise area, direction, method, and type from the controller home screen |
| **Task 2. Length and position setting** | |
| Sub-task 4 | Unlock the main unit and check the lock menu on the controller display |
| Sub-task 5 | Use a tape measure to measure calf and thigh lengths |
| Sub-task 6 | Input the calf and thigh measurements into the controller |
| Sub-task 7 | Load the affected area of the imaginary patient on the main unit |
| Sub-task 8 | Lock the main unit and check the lock menu on the controller display |
| Sub-task 9 | Set the initial position angle |
| **Task 3. Active exercise** | |
| Sub-task 10 | Set the angle range for exercise (flexion-30, extension-110), exercise intensity (flexion-15, extension-15), and duration of exercise (20 min) |
| Sub-task 11 | Start the exercise and pause after 3 min |
| Sub-task 12 | Restart the exercise and finish after 3 min |
| Sub-task 13 | Check the exercise record screen and return to the initial angle |
| **Task 4. Passive exercise** | |
| Sub-task 14 | Select the item area (knee), direction (right), method (passive), and type (exercise) from the tablet home screen |
| Sub-task 15 | Reset the length and position |
| Sub-task 16 | Set the angle range for the exercise (flexion-30, extension-110), wait time (10 s), exercise speed (5), and duration of exercise (20 min) |
| Sub-task 17 | Start the exercise and pause after 3 min |
| Sub-task 18 | Restart the exercise and finish after 3 min |
| Sub-task 19 | Check the exercise record screen and return to the initial angle |
| **Task 5. Active range of motion (ROM) measurement** | |
| Sub-task 20 | Select the item area (ankle), direction (left), method (active), and type (ROM measurement) from the tablet home screen |
| Sub-task 21 | Reset the length and position |
| Sub-task 22 | Start measuring the active ROM |
| Sub-task 23 | Finish measuring the active ROM |
| Sub-task 24 | Check the active ROM measurement results on the screen and return to the home screen |
| **Task 6. Passive ROM measurement** | |
| Sub-task 25 | Select the area (ankle), direction (left), method (passive), and type (ROM measurement) from the tablet home screen |
| Sub-task 26 | Reset the length and position |
| Sub-task 27 | Set the angle range for the exercise (flexion-30, extension-110, and speed–5) |
| Sub-task 28 | Start measuring the passive ROM |
| Sub-task 29 | Finish measuring the passive ROM |
| Sub-task 30 | Check the passive ROM measurement results on the screen and return to the home screen |
| **Task 7. Recorded exercise** | |
| Sub-task 31 | Select the item (direction–left and type–recorded exercise) from the tablet home screen |
| Sub-task 32 | After starting the recording, instruct the patient to move the device |
| Sub-task 33 | After completing the recording, edit the exercise portion to set it |
| Sub-task 34 | Start the recorded exercise |
| Sub-task 35 | Finish 3 min after starting the recorded exercise |
| Sub-task 36 | Check the exercise result screen |
| **Task 8. Turn off the power** | |
| Sub-task 37 | Turn the power off on the controller, then turn the power off on the main unit |
