# Supplementary material for: Usability Evaluation of an Electrically Powered Orthopedic Exerciser: Focus Group Interview and Satisfaction Survey Study
Source: JMIR Hum Factors. 2025 May 30;12:e60607. doi: 10.2196/60607 (PMC12143852; doi:10.2196/60607)
Supplement: Multimedia Appendix 2 [file humanfactors-v12-e60607-s002.docx]

Appendix 2. Contents Questions of Focus Group Interview

| Question process | Applications | Questions |
| --- | --- | --- |
| Opening  question | Beginning with the questions about general characteristics of the participants and questions related to interview topic  - To give all participants a chance to speak out | - General characteristics (age, gender, career, experience with lower limb rehabilitation devices, etc.)  - Are you currently providing treatment using lower limb rehabilitation devices such as electrically-powered orthopedic exerciser to your patients? |
| Transition  question | From the start question to the main question, the question helps the conversation move smoothly in the interview | - Could you tell me about your experience using lower extremity rehabilitation devices such as electrically-powered orthopedic exerciser for treatment?  - What types of treatment have you provided using electrically-powered orthopedic exerciser? (Treatment area/ROM angle/Passive, active, etc.)  - What do you think are the advantages of providing treatment using passive and active lower extremity rehabilitation devices?  - What should be considered when developing passive and active lower extremity rehabilitation devices that can be provided for treatment? |
| Key question | Through these questions, participants talked about their own opinions and experiences without a time limit  It consisted of questions about rebless pro, and an interview was conducted after providing a product demonstration. | - Do you have any comments about the user manual?  - Do you have any comments about the appearance and composition of the product?  - Are there any expected errors or risk factors when using the product? Or are there any areas that need improvement?  - Do you have any additional comments about rebless pro? |
| Ending question | Summarize the opinions collected in the discussion and see if there are anything missing or needed to be add | - Do you agree with this summary?  - Please let us know if you have any additional thoughts on what should be considered when developing an electrically-powered orthopedic exerciser. |
