# Supplementary material for: Usability Evaluation of an Electrically Powered Orthopedic Exerciser: Focus Group Interview and Satisfaction Survey Study
Source: JMIR Hum Factors. 2025 May 30;12:e60607. doi: 10.2196/60607 (PMC12143852; doi:10.2196/60607)
Supplement: Multimedia Appendix 3 [file humanfactors-v12-e60607-s003.docx]

Appendix 3. Survey results on ease of use of UI

| No. | Survey item | Responses from physiatrists | | Responses from physical therapists | |
| --- | --- | --- | --- | --- | --- |
|  |  | M | SD | M | SD |
| 1 | Check the user manual | 4.6 | 0.55 | 3.8 | 0.45 |
| 2 | Check the exterior of the product | 4.6 | 0.55 | 3.6 | 1.14 |
| 3 | Connect the power cable plug to the main unit connector and press the “ON” sign on the power switch to turn on the device | 4.6 | 0.55 | 4.6 | 0.55 |
| 4 | Check the controller power and select “Start exercise now” | 4.6 | 0.55 | 4.4 | 0.89 |
| 5 | Select the exercise area, direction, method, and type from the controller home screen | 4.8 | 0.45 | 4.4 | 0.89 |
| 6 | Unlock the main unit and check the lock menu on the controller display | 4 | 1.00 | 3.8 | 1.30 |
| 7 | Use a tape measure to measure calf and thigh lengths | 4 | 0.71 | 3.2 | 1.30 |
| 8 | Input the calf and thigh measurements into the controller | 4.6 | 0.55 | 3.4 | 1.34 |
| 9 | Load the affected area of the imaginary patient on the main unit | 4.2 | 0.84 | 3.8 | 1.30 |
| 10 | Set the initial position angle | 4 | 0.71 | 3.4 | 0.89 |
| 11 | For active exercise, select “Start the test” | 4.6 | 0.55 | 4.2 | 0.84 |
| 12 | For active exercise, select “Start exercise on completion” | 4.6 | 0.55 | 4.2 | 0.84 |
| 13 | For active exercise, set the angle range for active exercise | 4.4 | 0.89 | 4.0 | 1.22 |
| 14 | For active exercise, set the pace for a single session | 4.2 | 0.84 | 4.2 | 0.84 |
| 15 | For active exercise, set the exercise intensity | 4 | 0.71 | 4.4 | 0.55 |
| 16 | For active exercise, set the duration of exercise | 4.6 | 0.55 | 4.4 | 0.55 |
| 17 | For active exercise, select “Start exercise” | 4.8 | 0.45 | 4.4 | 0.55 |
| 18 | For active exercise, select “Pause” | 4.6 | 0.55 | 4.4 | 0.55 |
| 19 | For active exercise, select “Finish exercise” | 4.8 | 0.45 | 4.4 | 0.55 |
| 20 | For active exercise, check the exercise result screen | 4.8 | 0.45 | 4.4 | 0.89 |
| 21 | For passive exercise, select the exercise area, direction, method, and type from the controller home screen | 4.6 | 0.55 | 4.4 | 0.89 |
| 22 | For passive exercise, set the length and position | 4.6 | 0.55 | 3.8 | 1.30 |
| 23 | For passive exercise, set the angle range for exercise | 4.4 | 0.89 | 4.2 | 0.84 |
| 24 | For passive exercise, set the wait time | 4.8 | 0.45 | 4.2 | 0.84 |
| 25 | For passive exercise, set the exercise speed | 4.6 | 0.55 | 4.2 | 0.84 |
| 26 | For passive exercise, set the duration of exercise | 4.6 | 0.55 | 4.2 | 0.84 |
| 27 | For passive exercise, select “Start exercise” | 5 | 0.00 | 4.2 | 0.84 |
| 28 | For passive exercise, select “Pause” | 4.8 | 0.45 | 4.2 | 0.84 |
| 29 | For passive exercise, select “Restart exercise” | 4.8 | 0.45 | 4.2 | 0.84 |
| 30 | For passive exercise, select “Finish exercise” | 5 | 0.00 | 4.2 | 0.84 |
| 31 | For passive exercise, check the exercise result screen | 4.8 | 0.45 | 4.4 | 0.89 |
| 32 | For active range of motion (ROM) measurement, select the exercise area, direction, method, and type from the controller home screen | 4.2 | 0.45 | 4.4 | 0.89 |
| 33 | For active ROM measurement, set the length and position | 4 | 0.71 | 3.4 | 1.52 |
| 34 | Start active ROM measurement | 3.6 | 1.14 | 4.0 | 1.00 |
| 35 | Finish active ROM measurement | 3.8 | 0.84 | 4.0 | 1.00 |
| 36 | For active ROM measurement, check the test result screen | 4.4 | 0.55 | 4.4 | 0.89 |
| 37 | For passive ROM measurement, select the exercise area, direction, method, and type from the controller home screen | 4.4 | 0.55 | 4.4 | 0.89 |
| 38 | For passive ROM measurement, set the length and position | 4.4 | 0.55 | 3.4 | 1.52 |
| 39 | For passive ROM measurement, set the angle range for exercise | 4.4 | 0.55 | 3.8 | 1.30 |
| 40 | Start passive ROM measurement | 4.4 | 0.55 | 4.0 | 1.00 |
| 41 | Finish passive ROM measurement | 4.4 | 0.55 | 4.0 | 1.00 |
| 42 | For passive ROM measurement, check the test result screen | 4.4 | 0.55 | 4.4 | 0.89 |
| 43 | For recorded exercise, select the exercise direction and type from the controller home screen | 4.4 | 0.55 | 4.2 | 0.84 |
| 44 | For recorded exercise, set the length and position | 4.4 | 0.55 | 4.2 | 0.84 |
| 45 | For recorded exercise, select “Start recording” | 4.4 | 0.55 | 4.2 | 0.84 |
| 46 | For recorded exercise, select “Finish recording” | 4.4 | 0.55 | 4.2 | 0.84 |
| 47 | For recorded exercise, edit the recorded exercise portion | 4.4 | 0.55 | 3.8 | 1.30 |
| 48 | For recorded exercise, set the duration of exercise | 4.4 | 0.55 | 4.2 | 0.84 |
| 49 | For recorded exercise, start and finish the exercise | 4.4 | 0.55 | 4.2 | 0.84 |
| 50 | Stop operating the main unit and controller | 4.4 | 0.55 | 4.4 | 0.55 |
| 51 | Press the “OFF” sign on the power switch to turn off the device | 4.6 | 0.55 | 4.6 | 0.55 |
| M: Mean, SD: Standard Deviation, No.: Number | | | | | |
