# Supplementary material for: Usability Evaluation of an Electrically Powered Orthopedic Exerciser: Focus Group Interview and Satisfaction Survey Study
Source: JMIR Hum Factors. 2025 May 30;12:e60607. doi: 10.2196/60607 (PMC12143852; doi:10.2196/60607)
Supplement: Multimedia Appendix 4 [file humanfactors-v12-e60607-s004.docx]

Appendix 4. Survey results on identifiability and understanding of information

| No. | Survey item | Responses from physiatrists | | Responses from physical therapists | |
| --- | --- | --- | --- | --- | --- |
|  |  | M | SD | M | SD |
| 1 | Were you able to clearly see the exterior markings on the product (e.g., label, button signs, etc.)? | 4.4 | 0.55 | 3.0 | 2.00 |
| 2 | When using the controller, were you able to check the operational status of the screen? | 4.6 | 0.55 | 4.4 | 0.89 |
| 3 | Was the user manual helpful? | 4.4 | 0.55 | 3.8 | 0.45 |
| M: Mean, SD: Standard Deviation, No., Number | | | | | |
